# Supplementary material for: Early anthropoid femora reveal divergent adaptive trajectories in catarrhine hind-limb evolution
Source: Nat Commun. 2019 Nov 8;10:4778. doi: 10.1038/s41467-019-12742-0 (PMC6838095; doi:10.1038/s41467-019-12742-0)
Supplement: Supplementary file 1 — Supplementary Information [file 41467_2019_12742_MOESM1_ESM.pdf]

Supplementary Information

Early anthropoid femora reveal divergent adaptive trajectories in catarrhine hind-limb evolution

Almécija et al.

Table of Contents

- **Supplementary Note 1 | Description of DPC 24466**………………………..3

- **Supplementary Figures**
  - Supplementary Fig. 1 | Three-dimensional coordinates used in this paper, as seen on a human left proximal femur in posterior (left) and anterior (right) views…………………………………………………..5
  - Supplementary Fig. 2 | Femoral shaft proportions in anthropoids…….6
  - Supplementary Fig. 3 | Barplot showing the amount of variance explained by each of the axes in the principal components analysis….7
  - Supplementary Fig. 4 | Differentiation of proximal femoral shape in catarrhines…………………………………………………………..8
  - Supplementary Fig. 5 | Shape analysis of extant anthropoid proximal femora (fossils plotted *post hoc*)……………………………………....9
  - Supplementary Fig. 6 | Phylogenetic sensitivity analysis for the evolutionary modeling……………………………………………..10
  - Supplementary Fig. 7 | Akaike Information Criterion (AIC) values versus number of different evolutionary regimes as inferred by 'surface'…………………………………………………………10
  - Supplementary Fig. 8 | Univariate evolutionary modelling…………11
  - Supplementary Fig. 9 | Comparisons of plesiomophic anthropoid femora…………………………………………………………...12

- **Supplementary Tables**
    - Supplementary Table 1 | Description of landmarks on the proximal femur used in this study…………………………………………...13
    - Supplementary Table 2 | Extant sample summary…………………...14
    - Supplementary Table 3 | List of fossil specimens used in the shape and/or evolutionary analyses………………………………………16
    - Supplementary Table 4 | Neck-shaft angle analysis sample composition…………………………………………………………..17
    - Supplementary Table 5 | Body size estimates………………………..18
- **Supplementary References**…………………………………………………19

Supplementary Note 1 | Description of DPC 24466

All reported values are in mm (unless indicated) from the original femur using Mitutoyo digital calipers. Angular measures were made with a standard protractor. Circumference around the midshaft was measured with a tape measure.

This right femur is complete except for the distal portion, which is broken off at the shaft proximal to the epicondyles. There are two prominent transverse cracks, but all three pieces perfectly conjoin. The proximal crack runs from the base of the lesser trochanter to the third trochanter, and the more distal crack runs at approximately midshaft. The femoral head is somewhat abraded on the anterior and medial sides, exposing some cancellous bone. Subchondral bone is still visible on the femoral head. Otherwise the partial femur is well preserved and free of distortion. The preserved length of this specimen is 112.1 mm. Given the uncertainty about where the distal end would have terminated, it is not possible to reconstruct the original length of the femur, but it appears to have been fairly short.

Aside from the abrasion noted above, the proximal portion of the femur is well preserved. The dimensions of the femoral head are 13.2 mm superoinferiorly, 13.0 mm anteroposteriorly, and 11.4 mm deep on the anterior side. The dimensions of the femoral head would have been just slightly larger prior to abrasion. The fovea capitis is difficult to discern but is posteroinferior to the center of the subchondral surface. The femoral head is not conspicuously differentiated from the femoral neck (in terms of proximodistal diameter), and the articular surface is flush with the femoral neck superiorly and especially posteriorly. A tubercle is not present on the posterior femoral neck, as is typically seen in early hominoids (early and middle Miocene) and some other living [1] and extinct [2,3] anthropoids.

The neck is flattened anteroposteriorly. It measures 11.6 mm superoinferiorly and 6.3 mm at the narrowest point anteroposteriorly. The distance between the femoral head and the medial border of the greater trochanter, taken along the superior aspect of the femoral neck, is 6.4 mm. The biomechanical neck length is 22.1 mm. The femoral neck-shaft angle is 125º, and the neck is anteverted <15º.

The greater trochanter is intact but small pieces of cortical bone are missing at the medial apex of the greater trochanter and on the anterolateral surface. The trochanteric fossa is long and deep, and opens onto the posterior aspect of the femoral neck. The trochanteric fossa has a superoinferior length of about 14 mm and a maximum width of 3.6 mm. The intertrochanteric crest is weak as it passes towards the lesser trochanter. There is no anterior intertrochanteric line present.

The lesser trochanter is located 13.8 mm from the inferior border of the femoral head. The inferior portion is missing bone, but the preserved portion is approximately 10.7 mm superoinferiorly. The lesser trochanter is 7.4 mm mediolaterally, and 5.9 mm at its

narrowest point. It projects about 9 mm from the shaft in a slightly posteromedial direction. There is a gentle ridge that runs superiorly from the superior aspect of the lesser trochanter to blend into the posteroinferior femoral neck. The insertion for the caudal portion of the gluteus maximus is apparent as a prominent third trochanter, as previously noticed in other more fragmentary specimens [4].

Most of the femoral shaft is well preserved. The posterior proximal portion of the shaft preserves a keel that may correspond to the insertion of the adductor musculature, as inferred from modern monkeys [5]. Distally, the keel bifurcates into ridges that run to the medial and lateral sides of the distal shaft, potentially also serving as muscular attachment sites. There is slight weathering on the anterior side of the shaft, giving the bone a slightly porous texture anteriorly. The shaft is relatively straight in all views. The shaft is only slightly platymeric proximally but becomes highly platymeric closer to the approximate location of the midshaft (Supplementary Fig. 2). The proximal shaft dimensions are 11.3 mm mediolaterally and 10.5 mm anteroposteriorly. Although exact midshaft location cannot be determined due to the missing distal end, it could reasonably be inferred based on the contours of the shaft, which begins to flare distally for the femoral condyles. The dimensions at the approximate midshaft location are 12.1 mm mediolaterally and 10.9 mm anteroposteriorly, with a circumference of 37.4 mm. The distal shaft is 18.8 mm mediolaterally and 10.6 mm anteroposteriorly. The cortical bone exposed distally is thin, measuring <2 mm in all locations.

Supplementary Figures

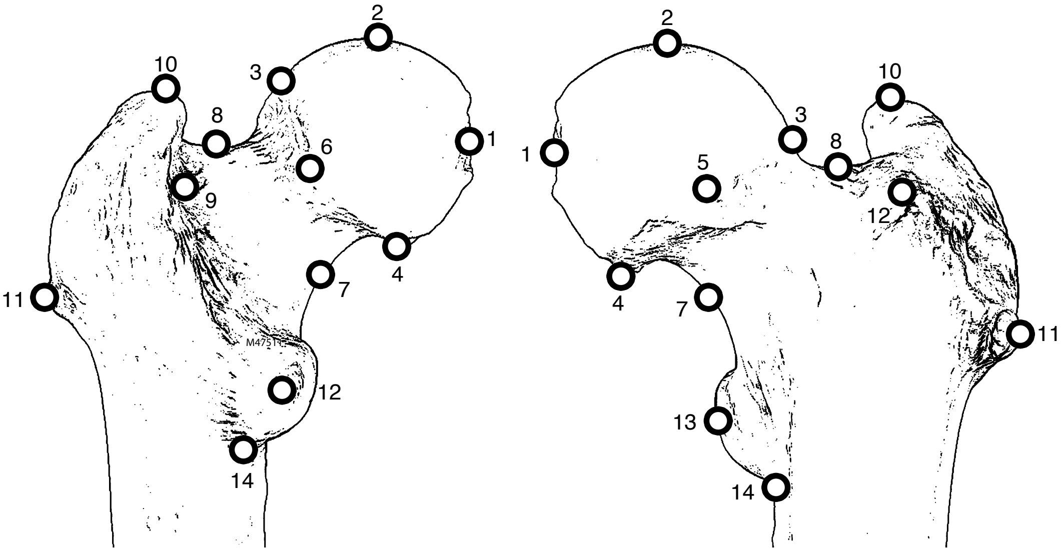

Supplementary Fig. 1 | Three-dimensional coordinates used in this paper, as seen on a human left proximal femur in posterior (left) and anterior (right) views. Points from the head and neck give an accurate depiction of the relative size of the head as well as height and length of the femoral neck. These traits have been shown to be related to the degree of mobility that is possible at the hip joint [6-9]. Points on the greater trochanter capture its maximal lateral and anterior projection, dimensions which probably reflect differences in gluteal function [6,10-12]. Finally, two points were collected on the lesser trochanter to quantify its relative size and the direction of its projection from the femoral shaft. The lesser trochanter is the insertion site for the iliopsoas muscle, the major flexor of the hip, and changes in lesser trochanter position partially reflect differences in iliopsoas lever arm length [6]. Coordinate (x, y, z) data were collected using a Microscribe 3DX digitizer by a single user to eliminate any effects of inter-observer error (MT). See Supplementary Table 1 for descriptions of each landmark.

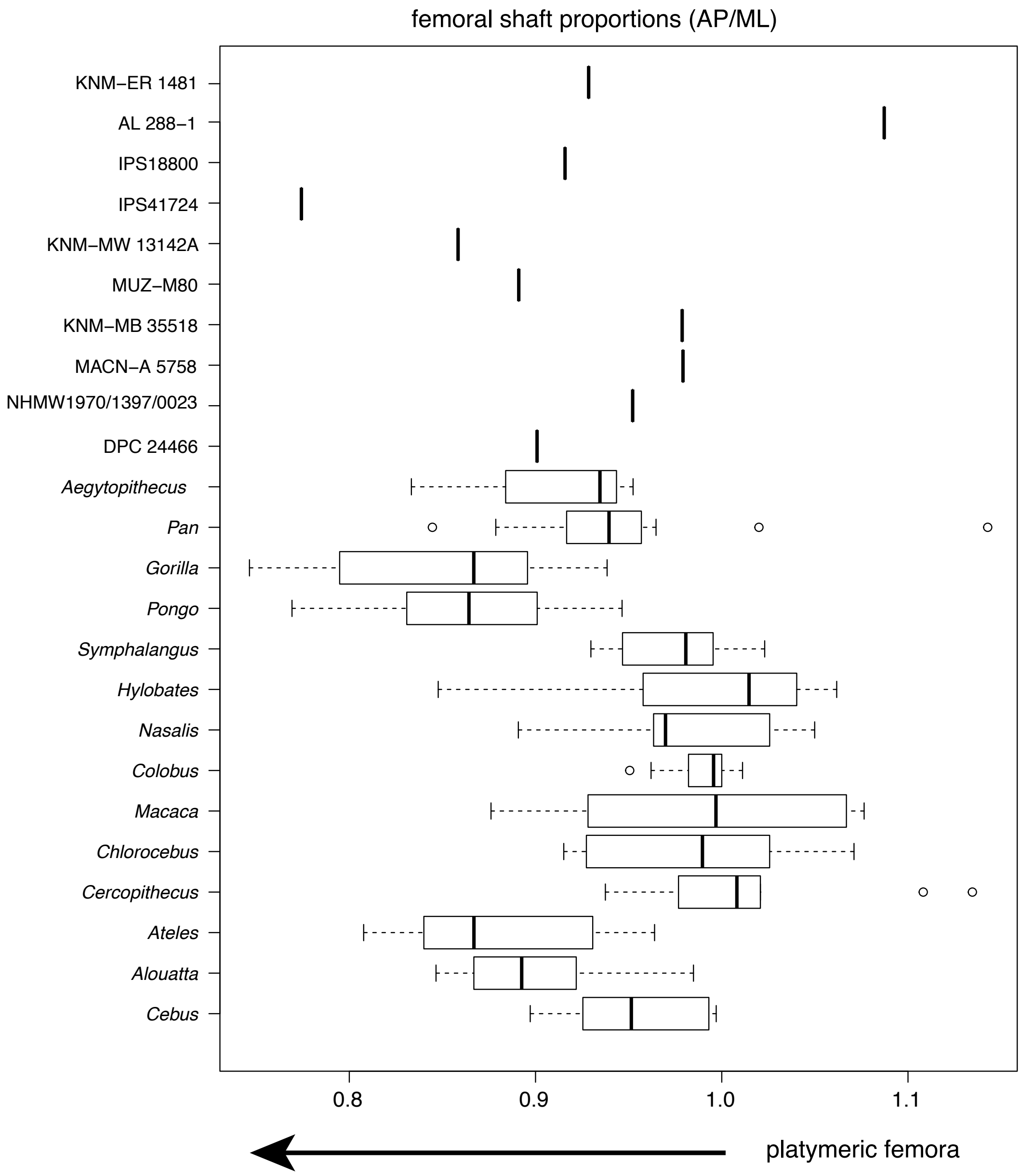

Supplementary Fig. 2 | Femoral shaft proportions in anthropoids. This measure was obtained as the ratio of anteroposterior-to-mediolateral midshaft diameters (AP and ML respectively). The ~0.9 value of the new *Aegyptopithecus* femur (DCP 24466) falls within the observed range of variation observed in previous specimens of this taxon ('*Aegyptopithecus*' boxplot). Boxplots represent 25th and 75th percentiles, respectively, centre line is the median, whiskers represent non-outlier range and dots are outliers. Extant sample composition is: *Alouatta palliata* (n=10), *Ateles fusciceps* (n=4), *Ateles geoffroyi* (n=4), *Ateles paniscus* (n=2), *Cebus apella* (n=10), *Cercopithecus mitis* (n=10), *Chlorocebus aethiops* (n=10), *Colobus guereza* (n=10), *Gorilla beringei beringei* (n=9), *Gorilla gorilla* (n=10), *Hylobates lar* (n=7), *Hylobates muelleri* (n=4), *Macaca fascicularis* (n=10), *Nasalis larvatus* (n=9), *Pan troglodytes* (n=16), *Papio anubis* (n=12), *Pongo abelii* (n=8), *Pongo pygmaeus* (n=14), *Symphalangus syndactylus* (n=10). The samples come from the collections of the

Museum of Comparative Zoology (MCZ), the National Museum of Natural History (NMNH), the Florida Museum of Natural History (FMNH), the Cleveland Museum of Natural History (CMNH) and the Bavarian Zoological Collection. The data is a combination of Ruff's data [13] with measurements taken by ASH. Measurements were taken with calipers in museums and from high-resolution 3D models in *PolyWorks* software. Source data are provided as a Source Data file.

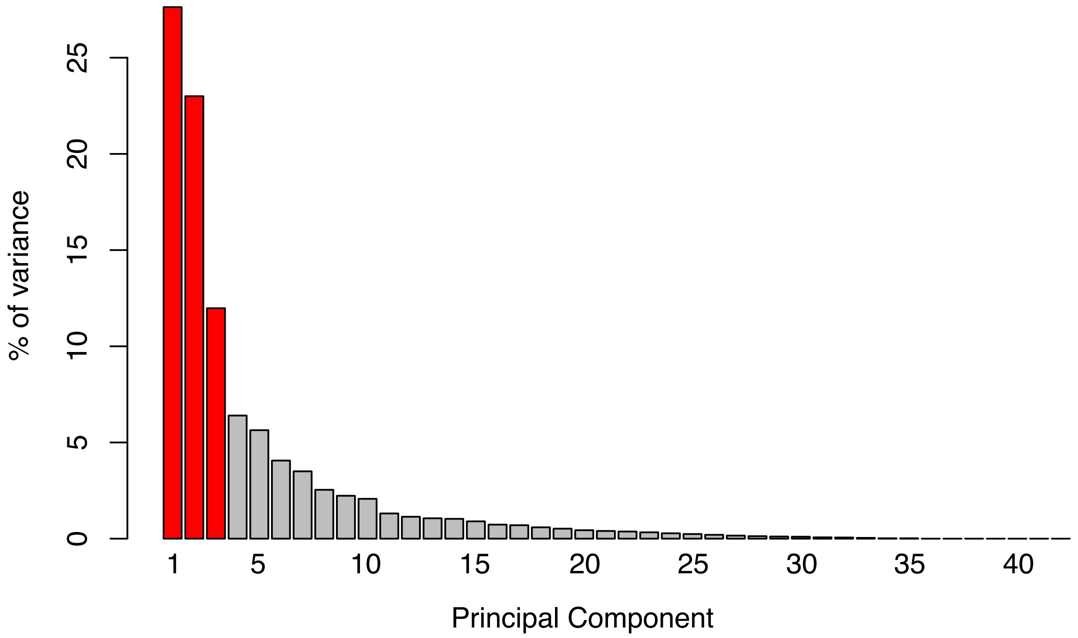

Supplementary Fig. 3 | Barplot showing the amount of variance explained by each of the axes in the principal components analysis. It can be difficult to decide how many components should be considered as important, although it is rare for more than the first two or three principal components to be easily interpretable (the rest are often considered 'noise'). For that reason, Hammer and Harper [14] suggest depicting the amount of explained variation as a descending curve called a 'scree plot' (or a barplot, as above), to assess where the eigenvalues start to flatten out. Beyond that point, the correlation between variables has been exhausted, and the components are probably not very informative. Following this criterion, the first three components (in red) were used in subsequent evolutionary analyses of anthropoid proximal femoral shape. This PCA was carried out on the covariance matrix of the total sample (using species means).

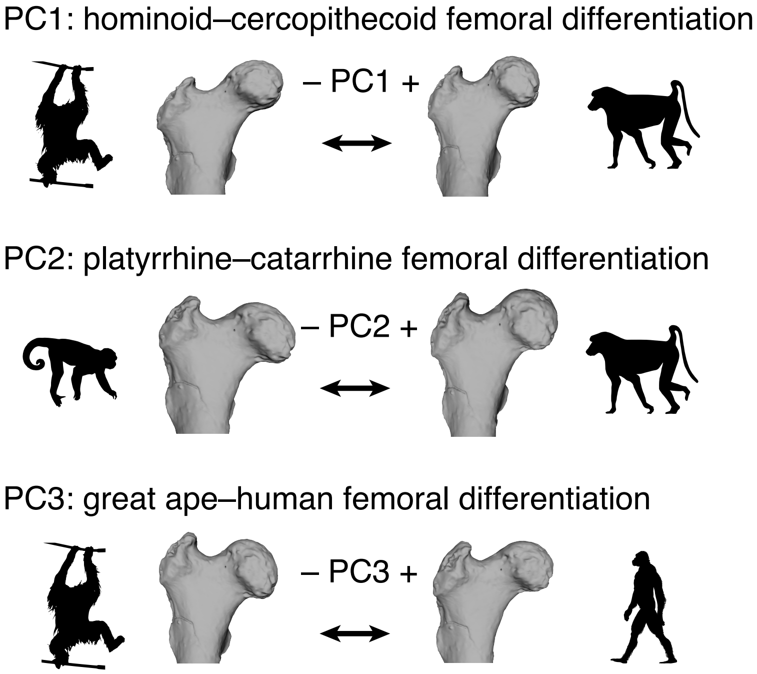

Supplementary Fig. 4 | Differentiation of proximal femoral shape in catarrhines. Based on the first principal component (PC1), platyrrhines and catarrhines (PC2) and great apes and humans (PC3) based on the analysis shown in Figure 3. Extreme morphologies along each axis are represented by thin-plate-spline (TPS) warped versions of DPC 24466. Silhouette for *Pongo* was custom made. Silhouettes for *Papio* and *Australopithecus* were downloaded from www.phylopic.org and is licensed for free use in the Public Domain without copyright. Silhouette for *Cebus apella* was also downloaded from www.phylopic.org (credit to Sarah Werning, and available for use under CC BY 3.0 license).

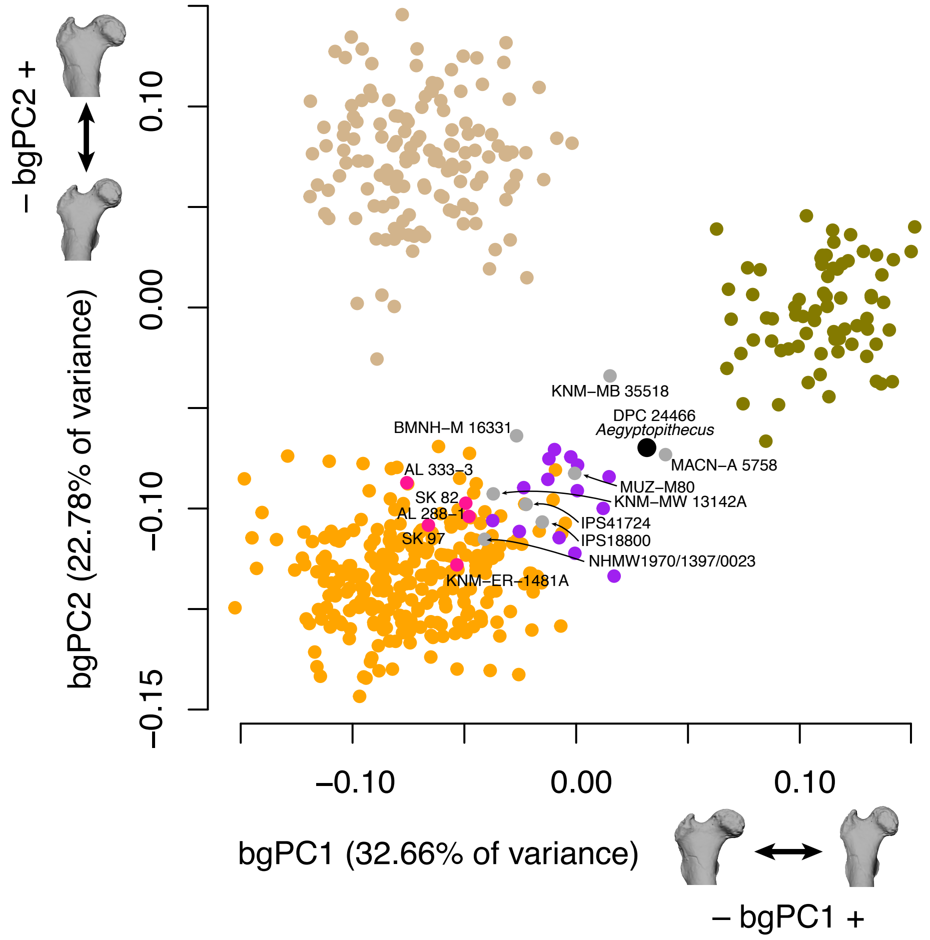

Supplementary Fig. 5 | Shape analysis of extant anthropoid proximal femora (fossils plotted *post hoc*). The plot shows the first two principal components of an analysis carried out on the between group covariance matrix (bgPCA). In this case, the groups represent only extant species centroids, with individual specimens and fossils plotted *post hoc*. Thin-plate-spline (TPS) warped versions of DPC 24466 depicting extremes of variation are presented for each axis. The color codes are as follow: New World monkeys, light brown; Old World monkeys, green; great apes and humans, orange; hylobatids, purple; fossil hominins, pink; other fossil primates, grey; the *Aegyptopithecus* DPC 24466 femur is black. Taxonomic attributions of the fossils represented are: DPC 24466 *Aegyptopithecus zeuxis*, MACN-A 5758 *Homunculus patagonicus*, KNM-MB 35518 *Victoriapithecus macinnesi*, NHMW1970/1397/0023 *Epipliopithecus vindobonensis*, MUZ-M80 *Morotopithecus bishopi*, KNM-MW 13142A *Ekembo nyanzae*, BMNH-M 16331 *Equatorius africanus*, IPS41724 cf. *Dryopithecus fontani*, IPS18800 *Hispanopithecus laietanus*, AL333-3 and AL288-1 *Australopithecus afarensis*, SK 82 and SK 97 cf. *Paranthropus robustus*, KNM-ER 1481 cf. *Homo erectus*. Source data are provided as a Source Data file.

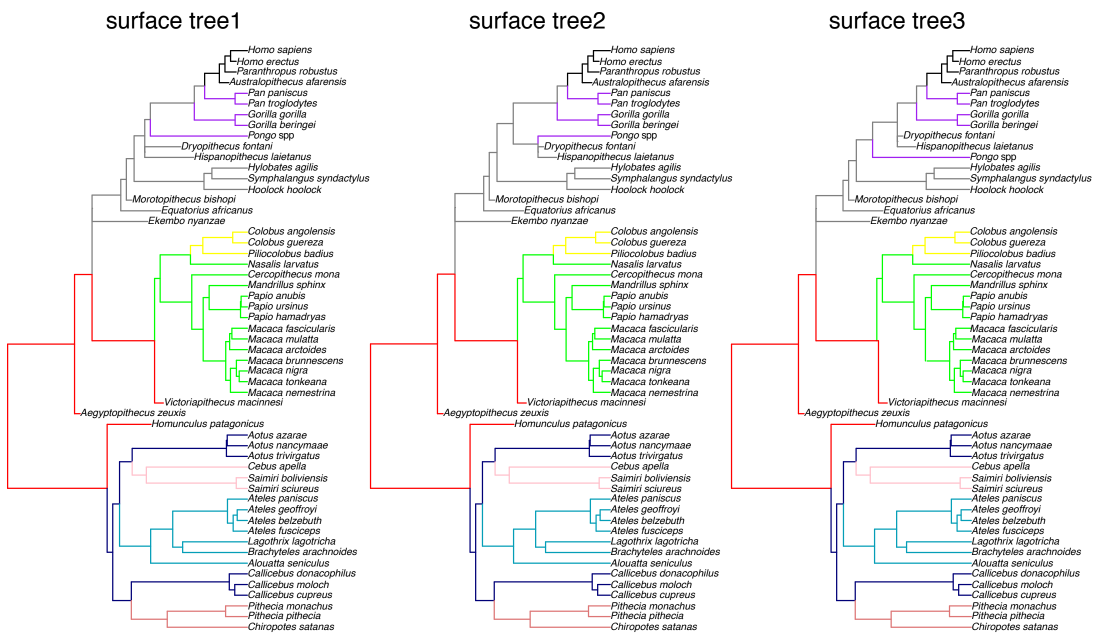

Supplementary Fig. 6 | Phylogenetic sensitivity analysis for the evolutionary modeling. The 'surface' output is compared under three hypothetical evolutionary scenarios: Catalan apes as stem great apes (tree1), stem pongines (tree2) and stem hominines (African apes and humans) (tree3). The results are exactly the same in all trees.

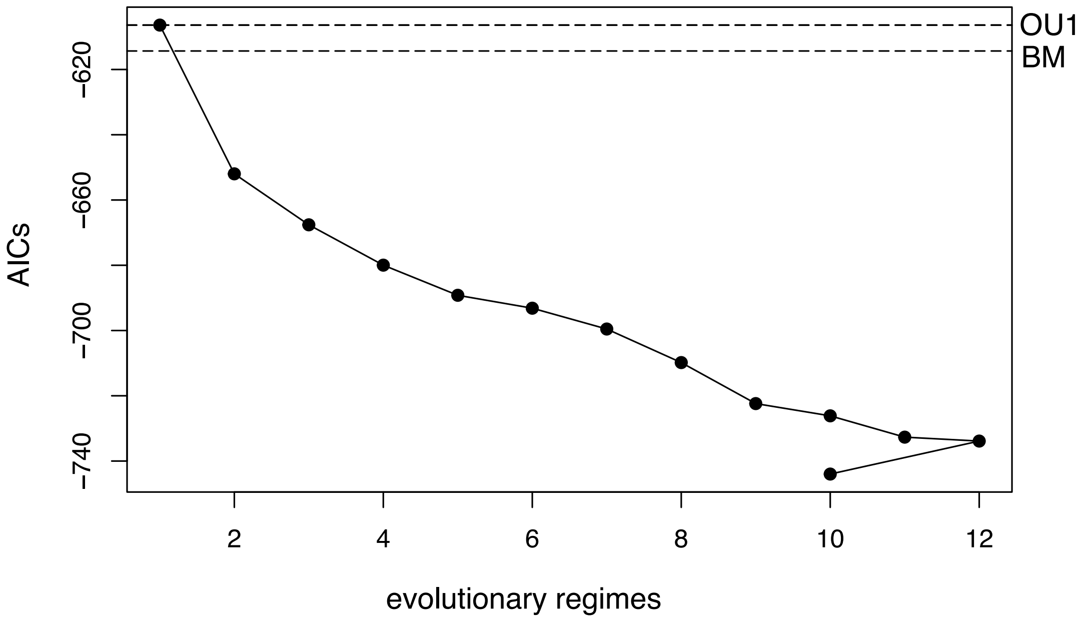

Supplementary Fig. 7 | Akaike Information Criterion (AIC) values versus number of different evolutionary regimes as inferred by 'surface'. Results are compared to standard Brownian motion (BM) and a single Ornstein-Uhlenbeck process (OU1).

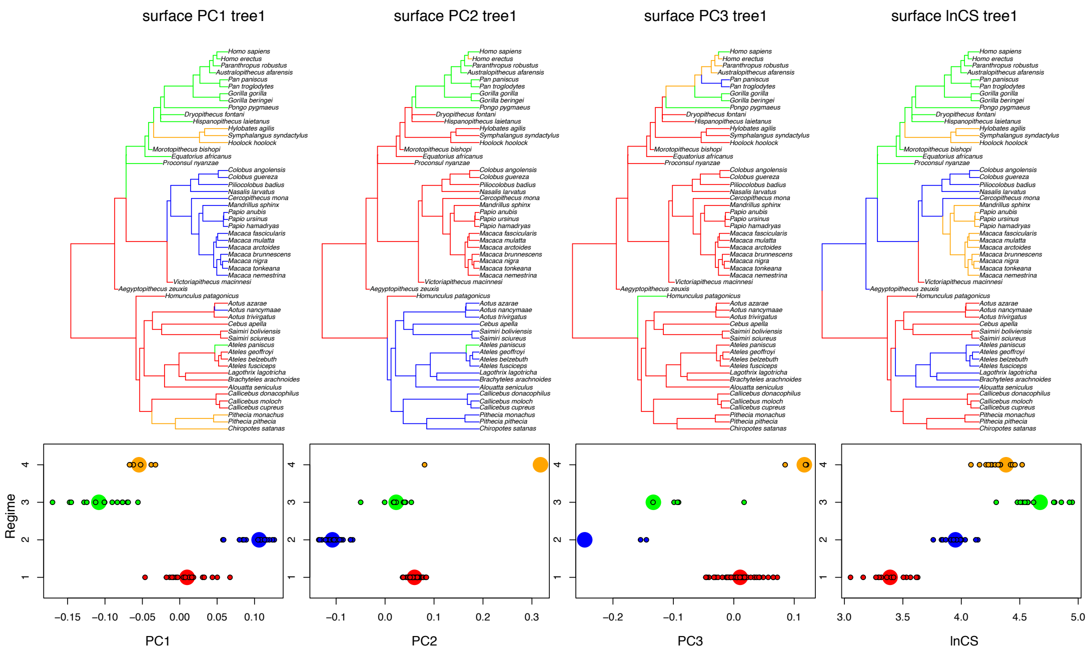

Supplementary Fig. 8 | Univariate evolutionary modelling. The 'surface' output is compared separately for each individual variable enlisted in the multivariate OU modelling: principal components (PC) 1-3 plus the ln-transformed centroid size (CS). The evolutionary optima detected by 'surface' in each case are shown in a chronometric phylogenetic tree (top panels) and as continuous trait space (bottom panels). The latter morphospaces show the estimated adaptive optima (large circles) and species (small circles) evolving under each evolutionary regime. The evolutionary regimes detected conform to some degree with the apparent morphological shifts among the major clades (Figs. 2,3 Supplementary Fig. 4): PC1 hominoids-cercopithecoids, PC2 platyrrhines-catarrhines, PC3 great apes-humans. Optima inferred outside the range of the actual range of data could reflect incomplete evolution towards distant optima. It could also represent a biologically unrealistic result derived from some of the model assumptions (e.g., keeping 'α' constant across the tree) [15]. In general, caution is advised when attempting to interpret the biological meaning of evolutionary regimes inferred from single traits, as it has been shown that the power of the analysis is lower, probably due to exceeding incidental convergence in low-dimensional trait space [15,16].

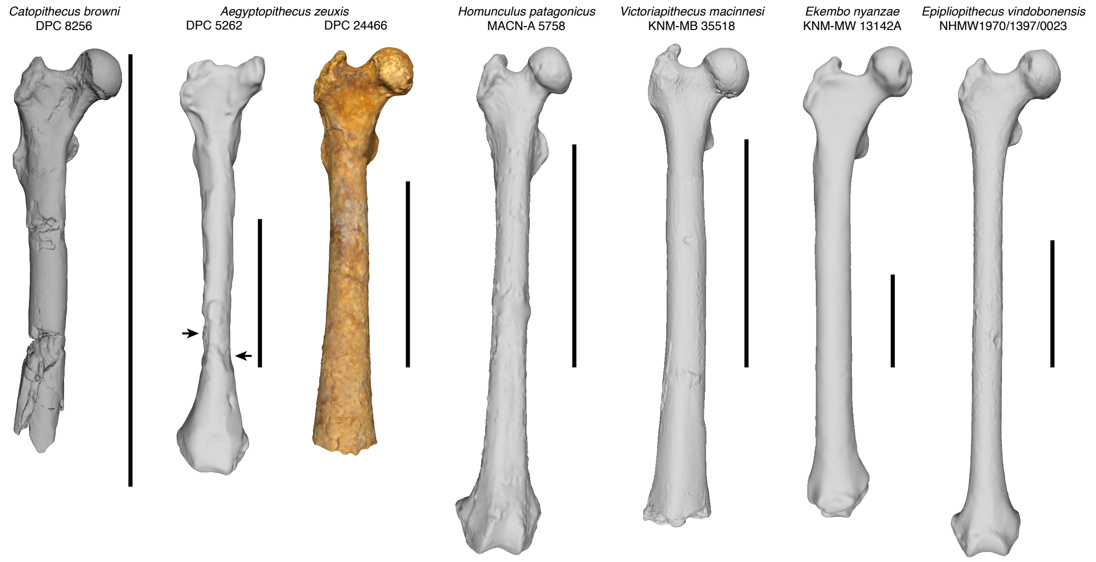

Supplementary Fig. 9 | Comparisons of plesiomophic anthropoid femora. The newly described femur of the stem catarrhine *Aegyptopithecus* (DPC 24466) is compared to the most complete previously known femur of this taxon (DPC 5262), the earlier possible stem catarrhine *Catopithecus* [17] as well as to that of the early New World monkey (NWM) *Homunculus*, an early Old World monkey (OWM) *Victoriapithecus*, a basal crown catarrhine (*Ekembo*), and a pliopithecoid (*Epipliopithecus*). The results of this study indicate that the proximal femora of *Aegyptopithecus* represent a plesiomorphic morphology which is not OWM-like nor hominoid-like, although it is similar to *Catopithecus*, but with a reduced third trochanter. The stem NWM *Homunculus* and the stem OWM *Victoriapithecus* apparently preserve this plesiomorphic morphology but *Ekembo* and *Epipliopithecus* have departed from it. The results of this study also suggest that some aspects of extant OWM proximal femoral morphology are as derived as in hominoids, but in a different direction (Fig. 3, Supplementary Fig. 4). Some of these morphologies are not captured by our metrics, but are already seen in *Victoriapithecus* (e.g., absence of third trochanter/gluteal tuberosity, morphology of the proximal portion of the greater trochanter). Arrows in DPC 5262 indicate areas where the shaft is damaged and some portions of cortical bone are missing. DPC 8256 and other available *Catopithecus* specimens could not be incorporated into the shape analyses because the proximal portions are anteroposteriorly flattened. Comparisons are based on 3D renderings of polygonal models scaled to similar mediolateral diameter. Scale bar represents 5 cm.

Supplementary Tables

Supplementary Table 1 | Description of landmarks on the proximal femur used in this study.

Number	Description
1	Middle of *fovea capitis femoris**
2	Most proximal point on the femoral head
3	Most proximal point on the facet margin
4	Most distal point of the facet margin
5	Most anterior point of the facet margin
6	Most posterior point of the facet margin
7	Maximum point of constriction on ridge running from lesser trochanter to the femoral head
8	Deepest point of the proximal neck
9	Deepest point of the trochanteric fossa
10	Most proximal point of the greater trochanter
11	Most lateral point of greater trochanter
12	Proximomedial extension of the greater trochanter on the anterior aspect of the femur
13	Tip of lesser trochanter
14	Most distal point of the lesser trochanter

* In *Pongo*, centre of the femoral head as in Harmon [18]. All these landmarks are type II/III following Bookstein [19].

Supplementary Table 2 | Extant sample composition summary (*N* = 502) used for the three-dimensional shape analyses and evolutionary modelling.

Hominoids	species	total
Homo sapiens **(n=97)**	*Homo sapiens*	97
Pan **(n=87)**	*Pan pansicus*	16
	Pan troglodytes	71
Gorilla **(n=77)**	*Gorilla beringei*	27
	Gorilla gorilla	50
Pongo **(n=16)**	*Pongo* spp	16
Hoolock **(n=9)**	*Hoolock hoolock*	9
Hylobates **(n=2)**	*Hylobates agilis*	2
Symphalangus **(n=3)**	*Symphalangus syndactylus*	3
**Old World monkeys**		
Cercopithecus **(n=7)**	*Cercopithecus mona*	7
Macaca **(n=24)**	*Macaca arctoides*	1
	Macaca brunnescens	4
	Macaca fascicularis	5
	Macaca mulatta	1
	Macaca nemestrina	5
	Macaca nigra	1
	Macaca tonkeana	7
Papio **(n=10)**	*Papio anubis*	2
	Papio hamadryas	2
	Papio ursinus	6
Mandrillus **(n=6)**	*Mandrillus sphinx*	6
Nasalis **(n=8)**	*Nasalis larvatus*	8
Colobus **(n=8)**	*Colobus angolensis*	4
	Colobus guereza	4
Piliocolobus **(n=8)**	*Piliocolobus badius*	8
**New World monkeys**		
Alouatta **(n=30)**	*Aloutta seniculus*	30
Ateles **(n=9)**	*Ateles geofroyii*	4
	Ateles fusciceps	2
	Ateles paniscus	1
	Ateles belzebuth	2
Brachyteles **(n=1)**	*Brachyteles arctoides*	1
Lagothrix **(n=2)**	*Lagothrix lagotricha*	2
Callicebus **(n=12)**	*Callicebus cupreus*	3
	Callicebus donacophilius	8
	Callicebus moloch	1
Chiropotes **(n=2)**	*Chiropotes satanas*	2
Pithecia **(n=4)**	*Pithecia pithecia*	1
	Pithecia monachus	3
Aotus **(n=30)**	*Aotus trivirgatus*	1
	Aotus nancymaae	2
	Aotus azarae	27
Cebus **(n=30)**	*Cebus apella*	30
Saimiri **(n=20)**	*Saimiri sciureus*	4
	Saimiri boliviensis	16
**TOTAL**		**502**

Landmark data were collected only on adult specimens with full epiphyseal closure. Extant non-human taxa were all wild-shot primates.

Individual specimen collection, access number and data are available through the 'figshare' repository (DOI 10.6084/m9.figshare.9461459), and in the Source Data file.

Supplementary Table 3 | List of fossil specimens used in the shape and/or evolutionary analyses.

Access number	Taxon	Point age estimates in tree
DPC 24466	*Aegyptopithecus zeuxis*	29.85 Ma
MACN-A 5758	*Homunculus patagonicus*	17.2 Ma
MUZ-M80	*Morotopithecus bishopi*	20.6 Ma
NHMW1970/1397/0023	*Epipliopithecus vindobonensis*	15 Ma
KNM-MW 13142A	*Ekembo nyanzae*	17.8 Ma
BMNH-M 16331	*Equatorius africanus*	15.47 Ma
KNM-MB 35518	*Victoriapithecus macinnesi*	15 Ma
IPS41724	cf. *Dryopithecus fontani*	11.9 Ma
IPS18800	*Hispanopithecus laietanus*	9.6 Ma
AL 333-3	*Australopithecus afarensis*	3.3 Ma
AL 288-1	*Australopithecus afarensis*	3.3 Ma
KNM-ER 1481	cf. *Homo erectus*	2 Ma
SK 82	*Paranthropus robustus*	2 Ma
SK 97	*Paranthropus robustus*	2 Ma

Only adult femora with complete and undistorted proximal portions were included in the analyses. The femur of *Epipliopithecus vindobonensis* was incorporated in the shape analyses (Fig. 3, Supplementary Figs. 3 and 8), but not in the evolutionary analyses due to its unresolved phylogenetic placement within Catarrhini. However, its impact in the results is discussed in the 'Discussion' section of the main text. See 'Methods' section in the main text for age estimates. Individual specimen information and data are available through the 'figshare' repository (DOI 10.6084/m9.figshare.9461459).

Supplementary Table 4 | Neck-shaft angle analysis sample composition.

species	mean	min	max	reference
Pan troglodytes (*n*=7)	122	115	130	MacLatchy et al. 2000. Ref. [20]
Gorilla beringei (*n*=7)	124	120	128	this study
Gorilla gorilla (*n*=10)	126	120	131	this study
Pongo spp (*n*=4)	145	135	152	MacLatchy et al. 2000. Ref. [20]
Symphalangus syndactylus (*n*=12)	134	130	138	this study
Hylobates lar (*n*=6)	129	125	133	MacLatchy et al. 2000. Ref. [20]
Hylobates muelleri (*n*=5)	134	130	140	MacLatchy et al. 2000. Ref. [20]
Hylobates concolor (*n*=7)	141	135	145	MacLatchy et al. 2000. Ref. [20]
Papio anubis (*n*=10)	126	120	130	this study
Cercopithecus neglectus (*n*=3)	117	114	121	Dagosto & Schmid 1996. Ref.[3]
Colobus sp (*n*=10)	121	117	125	Rose et al. 1992. Ref. [21]
Presbytis obscura (*n*=5)	119	116	120	Dagosto & Schmid 1996. Ref.[3]
Ateles sp (*n*=10)	140	130	150	Rose et al. 1992. Ref. [21]
Cebus sp (*n*=10)	123	110	135	Rose et al. 1992. Ref. [21]
Saimiri sciureus (*n*=4)	119	112	124	Dagosto & Schmid 1996. Ref.[3]
Pithecia pithecia (*n*=5)	125	122	131	Dagosto & Schmid 1996. Ref.[3]
Aegytopithecus zeuxis (DPC 5262)	--	120	130	Ankel-Simons et al. 1998. Ref.[4]
Aegytopithecus zeuxis (DPC 24466)	125			this study
Catopithecus browni (DPC 8256)	<125			this study
Epipliopithecus vindobonensis (NHMW1970/1397/0023)	130			Rose et al. 1992. Ref. [21]
Morotopithecus bishopi (MUZ-M80)	135			MacLatchy et al. 2000. Ref. [20]
Ekembo nyanzae (KNM-MW 13142A)	134			Ward et al. 1993. Ref. [22]
Equatorius africanus (BMNH 16331)	125			MacLatchy et al. 2000. Ref. [20]
Hispanopithecus laietanus (IPS18800)	132			Moyà-Solà et al. 2009. Ref. [23]
cf *Dryopithecus fontani* (IPS41724)	123			Moyà-Solà et al. 2009. Ref. [23]
Australopithecus afarensis (A.L. 288-1)	120			this study
Australopithecus robustus (SK 82)	115			this study
Australopithecus robustus (SK 97)	118			this study
cf *Homo erectus* (KNM-ER 1481)	120			this study
Homunculus patagonicus (MACN-A 5758)	125			this study
Victoriapithecus macinessi (KNM-MB 35518)	120			this study

The extant sample of 'this study' come the following collections: *Symphalangus syndactylus* (National Museum of Natural History, NMNH; Naturalis; Bavarian Zoological collection), *Gorilla beringei* (NMNH; Museum of Comparative Zoology, MCZ), *Gorilla gorilla* (NMNH; Cleveland Museum of Natural History, CMNH), *Papio anubis* (NMNH). Angular measurements from 'this study' were taken from high-resolution 3D models in *Fiji*. Individual source data are provided as a Source Data file.

Supplementary Table 5 | Body size estimates.

dimension	sample	estimate (kg)	upper 95%	lower 95%
femoral head SI	total sample	4.5	6.1	3.3
femoral head SI	hominoid sample	3.1	3.9	2.5
femoral head SI	cercopithecid sample	5	6.8	3.7
midshaft AP	total sample	6.8	9.3	4.9
midshaft AP	hominoid sample	8.5	13.2	5.5
midshaft AP	cercopithecid sample	7.1	9.4	5.4

Abbreviations: SI (supero-inferior diameter), AP (antero-posterior diameter). Regression are described in Ruff [24].

Supplementary References

1 Hammond, A. S. The anthropoid crista trochanterica and the hip joint capsule. *Anat. Rec.* **299**, 60-69, (2016).

2 Gebo, D. L., Simons, E. L., Rasmussen, T. D. & Dagosto, M. in *Anthropoid origins.* 203-233 (Plenum Press, 1994).

3 Dagosto, M. & Schmid, P. Proximal femoral anatomy of omomyiform primates. *J. Hum. Evol.* **30**, 29-56, (1996).

4 Ankel-Simons, F., Fleagle, J. G. & Chatrath, P. S. Femoral anatomy of *Aegyptopithecus zeuxis*, an Early Oligocene anthropoid. *Am. J. Phys. Anthropol.* **106**, 413-424, (1998).

5 Grand, T. I. The functional anatomy of the lower limb of the howler monkey (*Alouatta caraya*). *Am. J. Phys. Anthropol.* **28**, 163-181, (1968).

6 Aiello, L. & Dean, C. in *An Introduction to Human Evolutionary Anatomy* 457-482 (Academic Press, 1990).

7 MacLatchy, L. M. Another look at the australopithecine hip. *J. Hum. Evol.* **31**, 455-476, (1996).

8 Anderson, J. Y. & Trinkaus, E. Patterns of sexual, bilateral and interpopulational variation in human femoral neck-shaft angles. *J. Anat.* **192**, 279-285, (1998).

9 Ruff, C. Hindlimb articular surface allometry in hominoidea and *Macaca*, with comparisons to diaphyseal scaling. *J. Hum. Evol.* **17**, 687-714, (1988).

10 Lovejoy, O. C. The evolution of human walking. *Scientific American* **259**, 118-125, (1988).

11 Lovejoy, C. O., Heiple, K. G. & Burstein, A. H. The gait of *Australopithecus*. *Am. J. Phys. Anthropol.* **38**, 757-779, (1973).

12 Lovejoy, C. O., Meindl, R. S., Ohman, J. C., Heiple, K. G. & White, T. D. The Maka femur and its bearing on the antiquity of human walking: applying contemporary concepts of morphogenesis to the human fossil record. *Am. J. Phys. Anthropol.* **119**, 97-133, (2002).

13 Ruff, C. B. Long bone articular and diaphyseal structure in Old World monkeys and apes. I: Locomotor effects. *Am. J. Phys. Anthropol.* **119**, 305-342, (2002).

14 Hammer, O. & Harper, D. *Paleontological data analysis.* (Blackwell Publishing, 2006).

15 Ingram, T. & Mahler, D. L. SURFACE: detecting convergent evolution from comparative data by fitting Ornstein - Uhlenbeck models with stepwise Akaike Information Criterion. *Methods Ecol. Evol.* **4**, 416-425, (2013).

16 Stayton, C. T. Is convergence surprising? An examination of the frequency of convergence in simulated datasets. *Journal of Theoretical Biology* **252**, 1-14, (2008).

17 Seiffert, E. R. Early primate evolution in Afro-Arabia. *Evol. Anthropol.* **21**, 239-253, (2012).

18 Harmon, E. H. The shape of the hominoid proximal femur: a geometric morphometric analysis. *J. Anat.* **210**, 170-185, (2007).

19 Bookstein, F. L. *Morphometric Tools for Landmark Data: Geometry and Biology.*, (University Press, 1991).

20 MacLatchy, L., Gebo, D., Kityo, R. & Pilbeam, D. Postcranial functional morphology of *Morotopithecus bishopi*, with implications for the evolution of modern ape locomotion. *J. Hum. Evol.* **39**, 159-183, (2000).

21 Rose, M. D., Leakey, M. G., Leakey, R. E. F. & Walker, A. C. Postcranial specimens of *Simiolus enjiessi* and other primitive catarrhines from the early Miocene of Lake Turkana, Kenya. *J. Hum. Evol.* **22**, 171-237, (1992).
22 Ward, C. V., Walker, A., Teaford, M. F. & Odhiambo, I. Partial skeleton of *Proconsul nyanzae* from Mfangano Island, Kenya. *Am. J. Phys. Anthropol.* **90**, 77-111, (1993).
23 Moyà-Solà, S. *et al.* First partial face and upper dentition of the Middle Miocene hominoid *Dryopithecus fontani* from abocador de Can Mata (Vallès-Penedès Basin, Catalonia, NE Spain): taxonomic and phylogenetic implications. *Am. J. Phys. Anthropol.* **139**, 126-145, (2009).
24 Ruff, C. B. Long bone articular and diaphyseal structure in Old World monkeys and apes. II: Estimation of body mass. *Am. J. Phys. Anthropol.* **120**, 16-37, (2003).
