# Supplementary material for: Early anthropoid femora reveal divergent adaptive trajectories in catarrhine hind-limb evolution
Source: Nat Commun. 2019 Nov 8;10:4778. doi: 10.1038/s41467-019-12742-0 (PMC6838095; doi:10.1038/s41467-019-12742-0)
Supplement: Supplementary file 3 — Reporting Summary [file 41467_2019_12742_MOESM3_ESM.pdf]

nature research

Corresponding author(s): Sergio Almécija

Last updated by author(s): Sep 16, 2019

Reporting Summary

Nature Research wishes to improve the reproducibility of the work that we publish. This form provides structure for consistency and transparency in reporting. For further information on Nature Research policies, see Authors & Referees and the Editorial Policy Checklist .

Statistics

For all statistical analyses, confirm that the following items are present in the figure legend, table legend, main text, or Methods section.

n/a	Confirmed	
☐	☒	The exact sample size (*n*) for each experimental group/condition, given as a discrete number and unit of measurement
☐	☒	A statement on whether measurements were taken from distinct samples or whether the same sample was measured repeatedly
☒	☐	The statistical test(s) used AND whether they are one- or two-sided<br>*Only common tests should be described solely by name; describe more complex techniques in the Methods section.*
☒	☐	A description of all covariates tested
☒	☐	A description of any assumptions or corrections, such as tests of normality and adjustment for multiple comparisons
☒	☐	A full description of the statistical parameters including central tendency (e.g. means) or other basic estimates (e.g. regression coefficient) AND variation (e.g. standard deviation) or associated estimates of uncertainty (e.g. confidence intervals)
☒	☐	For null hypothesis testing, the test statistic (e.g. *F*, *t*, *r*) with confidence intervals, effect sizes, degrees of freedom and *P* value noted<br>*Give P values as exact values whenever suitable.*
☒	☐	For Bayesian analysis, information on the choice of priors and Markov chain Monte Carlo settings
☐	☒	For hierarchical and complex designs, identification of the appropriate level for tests and full reporting of outcomes
☒	☐	Estimates of effect sizes (e.g. Cohen's *d*, Pearson's *r*), indicating how they were calculated

Our web collection on statistics for biologists contains articles on many of the points above.

Software and code

Policy information about availability of computer code

Data collection	3D phenotypic coordinate (x, y, z) data were collected using a Microscribe 3DX digitizer by a single user to eliminate any effects of inter-observer error.
Data analysis	All statistical analyses were performed using the open access R statistical environment.

For manuscripts utilizing custom algorithms or software that are central to the research but not yet described in published literature, software must be made available to editors/reviewers. We strongly encourage code deposition in a community repository (e.g. GitHub). See the Nature Research guidelines for submitting code & software for further information.

Data

Policy information about availability of data

All manuscripts must include a data availability statement. This statement should provide the following information, where applicable:

- Accession codes, unique identifiers, or web links for publicly available datasets
- A list of figures that have associated raw data
- A description of any restrictions on data availability

Three-dimensional digital data from the new Aegyptopithecus femur described in this study (DPC 24466) are available in MorphoSource's project 'Duke Lemur Center Division of Fossil Primates' (P114), under the media number M47511. Raw data used in all geometric morphometric and evolutionary analyses are available through the 'figshare' respository (https://figshare.com/s/ebbc90c136319f9ff2cf; DOI 10.6084/m9.figshare.9461459). Nexus trees used in the evolutionary modelling are also available as Supplementary Data 1–3. The source data underlying Figs. 2, 3b, 3c, Supplementary Figs. 2, 5 and Supplementary Table 4 are provided as a Source Data file.

Field-specific reporting

Please select the one below that is the best fit for your research. If you are not sure, read the appropriate sections before making your selection.

☐ Life sciences ☐ Behavioural & social sciences ☒ Ecological, evolutionary & environmental sciences

For a reference copy of the document with all sections, see nature.com/documents/nr-reporting-summary-flat.pdf

Ecological, evolutionary & environmental sciences study design

All studies must disclose on these points even when the disclosure is negative.

Study description	The study uses a series of stepwise analyses of a large sample of living and fossil anthropoid femora, combining three-dimensional geometric morphometrics (3DGM), and evolutionary modelling within a multi-regime, multivariate framework. Importantly, relevant fossils were incorporated in the analyses, including a new femur of Aegyptopithecus zeuxis, an early Oligocene advanced stem catarrhine from Egypt.<br><br>The results of this study show that cercopithecoids and hominoids have undergone divergent evolution of the hip complex (on the femoral side) from an ancestral morphology not present in living anthropoids, which is represented in Aegyptopithecus, stem platyrrhines, and stem cercopithecoids. Based on the 'intermediate' morphometric position identified for Aegyptopithecus and its inferred plesiomorphic evolutionary regime, the ancestor of cercopithecoids and hominiods was not specialised towards neither the distinct locomotor repertoires exhibited by both modern groups. These results highlight the relevance of fossil evidence for illuminating key adaptive shifts in primate evolution.
Research sample	This study incorporates a large sample of extant and fossil anthropoids (N= 502). It samples all living hominid species (great apes and humans), as well as representative genera/species of hylobatids, cercopithecoids (Old World monkeys) and platyrrhines (New World monkeys). The objective is to morphometrically characterize the phenetic affinities of a new Aegyptopithecus femur and to use the subsequent morphometric data to model the evolution of the hip joint after the hominoid-cercopithecoid divergence.
Sampling strategy	Evolutionary analyses rely on the number of taxa as sample size (n = 55 in this case). In this study, for each case, good taxonomic representation is ensured by using several (sometimes dozens) individuals to compute each species average –representing each taxon.
Data collection	Data was collected by a single author (LT) to avoid inter-observer error. Data were collected using a Microscribe 3DX digitizer.
Timing and spatial scale	Comparative sample was collected at regular intervals during a period of time of 3 months.
Data exclusions	No data were excluded from the analyses.
Reproducibility	Using the data provided, and following the methodological steps described in the study (and the same software), it will lead to exactly the same results. This was found in 5+ iterations of each analysis.
Randomization	For living taxa, individual specimens were allocated to their assigned species based on museum records.
Blinding	Blinding is not compatible with the approach of this study (i.e., the study relies on large inter-specific comparisons for which group allocation must be known a priori).

Did the study involve field work? ☐ Yes ☒ No

Reporting for specific materials, systems and methods

We require information from authors about some types of materials, experimental systems and methods used in many studies. Here, indicate whether each material, system or method listed is relevant to your study. If you are not sure if a list item applies to your research, read the appropriate section before selecting a response.

Materials & experimental systems

n/a	Involved in the study	
☒	☐	Antibodies
☒	☐	Eukaryotic cell lines
☐	☒	Palaeontology
☒	☐	Animals and other organisms
☒	☐	Human research participants
☒	☐	Clinical data

Methods

n/a	Involved in the study	
☒	☐	ChIP-seq
☒	☐	Flow cytometry
☒	☐	MRI-based neuroimaging

Palaeontology

Specimen provenance	Fieldwork in the Fayum (Egypt) area was facilitated by scientists from the Egyptian Geological Museum, the Egyptian Mineral Resources Authority, and the Egyptian Environmental Affairs Agency.
Specimen deposition	The fossil will be deposited for open study in the Duke Lemur Center (Duke University). 3D scans are available in MorphoSourece's project "Duke Lemur Center Division of Fossil Primates" (P114).
Dating methods	No new dates are provided.

☐ Tick this box to confirm that the raw and calibrated dates are available in the paper or in Supplementary Information.
