# Supplementary material for: Early anthropoid femora reveal divergent adaptive trajectories in catarrhine hind-limb evolution
Source: Nat Commun. 2019 Nov 8;10:4778. doi: 10.1038/s41467-019-12742-0 (PMC6838095; doi:10.1038/s41467-019-12742-0)
Supplement: Supplementary file 4 — Description of Additional Supplementary Files [file 41467_2019_12742_MOESM4_ESM.pdf]

Description of Additional Supplementary Files

File Name: Supplementary Data 1
Description: tree1 European apes as stem great apes. Nexus file.

File Name: Supplementary Data 2
Description: tree2 European apes as stem hominines. Nexus file.

File Name: Supplementary Data 3
Description: tree3 European apes as stem pongines. Nexus file.
